# Post-Processing Kalman Filter Application for Improving Cooperative Awareness Messages’ Position Data Accuracy

**DOI:** 10.3390/s24247892

**Published:** 2024-12-10

**Authors:** Maximilian Bauder, Robin Langer, Tibor Kubjatko, Hans-Georg Schweiger

**Affiliations:** 1 CARISSMA Institute of Electric, Connected and Secure Mobility, Technische Hochschule Ingolstadt, Esplanade 10, 85049 Ingolstadt, Germany; robin.langer@thi.de (R.L.); hans-georg.schweiger@thi.de (H.-G.S.); 2 Institute of Forensic Research and Education, University of Zilina, 010 26 Zilina, Slovakia; tkubjatko@gmail.com

**Keywords:** cooperative awareness message, CAM, C-ITS, Kalman filter, accident analysis, V2X, ETC service

## Abstract

Cooperative intelligent transportation systems continuously send self-referenced data about their current status in the Cooperative Awareness Message (CAM). Each CAM contains the current position of the vehicle based on GPS accuracy, which can have inaccuracies in the meter range. However, a high accuracy of the position data is crucial for many applications, such as electronic toll collection or the reconstruction of traffic accidents. Kalman filters are already frequently used today to increase the accuracy of position data. The problem with applying the Kalman filter to the position data within the Cooperative Awareness Message is the low temporal resolution (max. 10 Hz) and the non-equidistant time steps between the messages. In addition, the filter can only be applied to the data retrospectively. To solve these problems, an Extended Kalman Filter and an Unscented Kalman Filter were designed and investigated in this work. The Kalman filters were implemented with two kinematic models. Subsequently, driving tests were conducted with two V2X vehicles to investigate and compare the influence on the accuracy of the position data. To address the problem of non-equidistant time steps, an iterative adjustment of the Process Noise Covariance Matrix Q and the introduction of additional interpolation points to equidistance the received messages were investigated. The results show that without one of these approaches, it is impossible to design a generally valid filter to improve the position accuracy of the CAM position data retrospectively. The introduction of interpolation points did not lead to a significant improvement in the results. With the Q matrix adaptation, an Unscented Kalman Filter could be created that improves the longitudinal position accuracy of the two vehicles under investigation by up to 80% (0.54 m) and the lateral position accuracy by up to 72% (0.18 m). The work thus contributes to improving the positioning accuracy of CAM data for applications that receive only these data retrospectively.

## 1. Introduction

The direct communication of Cooperative Intelligent Transport Systems (C-ITS) is one of the most exciting future vehicle technologies, as it promises many improvements in safety, comfort, efficiency, and more [1,2]. There are vehicles on the European market today, most notably from Volkswagen, which already use this technology on the roads daily [3]. Direct communication is also referred to as Vehicle-2-Everything communication (V2X) and is divided into communication with vehicles (V2V), infrastructure (V2I), and, in the future, with people (V2P) [4]. Current vehicles continuously send their vehicle status in the Cooperative Awareness Message (CAM) and send an event-based Decentralized Environment Notification Message (DENM) [5,6].

The CAM contains the current position data of the transmitting vehicle and represents an interesting data source for future accident analysis [1]. Although digital data storage devices already exist, such as the Event Data Recorder following UNECE R160, no position data are stored in it today [7]. Precise position data are crucial for accurately and unambiguously reconstructing the course of the accident, and they would represent a significant improvement in the digital database for accident reconstruction. Therefore, in a previous study, we investigated the accuracy of all CAM data under various dynamic conditions (standing, accelerated driving, constant driving, cornering, etc.) [8].

As can be read in [8], we found a median error in position accuracy of between 0.64 m and 2.46 m in the longitudinal direction and from 0.29 m to 1.12 m in the lateral direction, depending on the dynamic driving state. The accuracy is, therefore, in the range of GPS accuracy and is often not even sufficient to meet the requirements of the safety-relevant V2X use cases [9,10]. The determined errors in the positional accuracy of the CAM could also result in difficulties for the plausible reconstruction of the course of the accident and the accident constellation at the time of contact.

To solve this problem, the use of Kalman filters and their extensions can often be found in the literature [11,12]. One major field of application is using Kalman filters for improving the accuracy of GPS position data [13,14,15,16]. Kalman filters are also frequently used to estimate system states. One example is the development of Kalman filters used for battery state of charge (SoC) estimation [17,18,19]. Another use case is vehicle state estimation. The position of the vehicle is fused from all available vehicle data sources (vehicle sensors, GPS, V2X, etc.) using a Kalman filter, which plays a crucial role in the realization of Advanced Driving Assistant Systems (ADAS) in particular [20,21,22,23]. However, none of these works have specifically designed a filter based solely on CAM data for post-processing to improve position accuracy.

In contrast, only a few studies could be found that dealt explicitly with the use of Kalman filters in combination with V2X communication [24,25,26,27,28]. Of these, only the authors of [27,28] deal with improving CAM position data through the use of Kalman filters.

The authors of [25,26] present methods for misbehavior detection of Cooperative Intelligent Transport Systems. Misbehavior detection is another application that requires high accuracy of the transmitted position. By verifying and checking the plausibility of the position of the transmitting vehicles, it is possible to identify misbehaving entities. Applying the Kalman filter, as described in [24], the predicted data from the Kalman filter and the actual position data received can be used to check whether the entities are moving as expected. However, the studies on misbehavior detection did not investigate how the Kalman filter improves the accuracy of the data. The data from the Kalman filter is only used to check the plausibility of the CAM position data sent.

In [26], the authors investigated the application of the Kalman filter to correct and predict the position of C-ITS. For this purpose, the authors used model-based and real vehicle kinematics data. The performance of the Kalman filter was also compared with that of an Artificial Neural Network (ANN). As a result, they found that the Kalman filter outperformed the ANN in improving position accuracy. In contrast to our study, only a linear Kalman filter approach was chosen. Furthermore, the real vehicle kinematics data are not extracted from actual CAMs but generated by additional measurement technology and GPS receivers. The parameterization and actual design of the Kalman filter also remains unclear in this work.

In contrast, the studies [27,28] deal more specifically with improving the CAM position data required to realize an Electronic Toll Collection (ETC) Service. The background is that each vehicle must be assigned to the correct lane to realize the ETC service. As in this work, the Kalman filter also processes the CAM data retrospectively. For this purpose, various kinematic models (Constant Turn Rate and Acceleration Model (CTRA), Constant Turn Rate and Velocity Model (CTRV), Constant Velocity Model (CV), and Constant Acceleration Model (CA)) were examined in the Kalman and Extended Kalman Filter [28]. It was shown that the Extended Kalman Filter with the CTRA model delivers the best results. However, The authors of [28] does not provide specific information on improving accuracy. It is also unclear which vehicles, V2X modules, and reference systems were used for the tests. The parameterization and actual design of the Kalman filter also remains unclear in this work.

None of the studies mentioned above considered and investigated the challenges of applying the Kalman filter to the CAM position data:

1. The data have a low temporal resolution between 1.0 and 10 Hz [5,29];
2. Time intervals between the CAMs are mostly non-equidistant, as they change with the change in the dynamic state of the V2X vehicle. This can lead to divergence of the filter, as the Process Noise Covariance Matrix (Q matrix) is not correct when the time step between the data changes [19].

Therefore, this work aims to design a Kalman filter to correct the CAM position data in post-processing to increase the accuracy for accident analysis and other applications. The designed filter should only have the CAM data available and demonstrate the applicability and feasibility of CAM data in post-processing. For this purpose, we designed an Extended Kalman Filter (EKF) and Unscented Kalman Filter (UKF) with the results of [8] and compared them based on two kinematic approaches.

Two approaches are investigated to solve the challenges: adapting the Q matrix and inserting additional interpolation points for artificial equidistance by linear interpolation between the CAMs.

The literature shows that only a few studies have dealt with the design of Kalman filters to improve CAM position data accuracy, especially when applied in post-processing. Moreover, the filter designed in this paper is based only on the information contained in the CAM. Furthermore, the difficulties mentioned above for Kalman filters have not yet been addressed and systematically investigated in the literature concerning CAM. In addition, this work is the first to design a Kalman filter based on real driving test data [8] with vehicles already on the market and to investigate its applicability and feasibility. The design and parameterization of the Kalman filter are also presented and can be used as a basis for further development and research for other work. To the best of our knowledge, all this represents a novelty within the literature. Thus, the main contribution of this work is the design of a Kalman filter that can process CAM data as a single data source to improve the accuracy of the position data the CAM contains. The results can significantly improve accident reconstruction with CAM data and other applications (e.g., ETC, misbehavior detection, V2X use cases, etc.).

## 2. Materials and Methods

### 2.1. Methodological Approach

**Figure 1:**
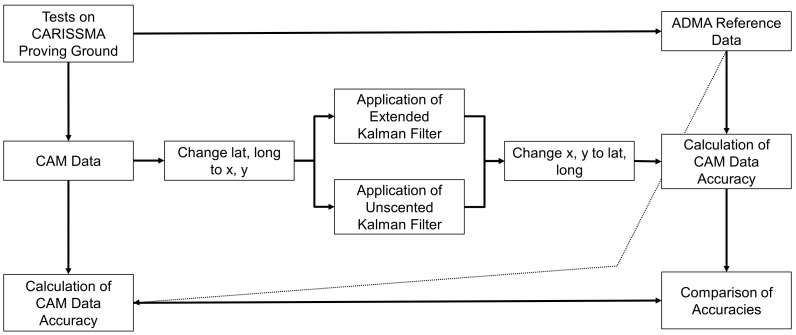
General methodological approach to investigate the Extended Kalman and Unscented Kalman Filter to improve the accuracy of the position information of the Cooperative Awareness Message.

In this work, an Extended Kalman Filter (EKF) and an Unscented Kalman Filter (UKF) were designed to retrospectively improve the accuracy of position information in Cooperative Awareness Messages (CAMs). The basic methodology is shown in Figure 1. Driving tests on the accuracy of CAMs were carried out in the CARISSMA test area [8]. This allowed both CAM and reference data to be collected using an ADMA-G Pro+ (Version 3.3.2) [19]. In [8], these data were used to determine the accuracy of the data in various driving scenarios. Based on the knowledge gained from this, the applicability of the EKF and UKF for improving position accuracy was investigated in this work. For this purpose, the filters were implemented in a Python script, and the position data were first transformed from latitude and longitude into global Cartesian coordinates. After applying the Kalman filters, the coordinates were then transformed back. The accuracies of the filtered position data $dx_{KF}$ and $dy_{KF}$ were then determined analogously to the procedure in [8] and compared with the accuracy of the position data without applying the Kalman filters ($dx_{CAM},dy_{CAM}$). The aim was to determine whether using Kalman filters can increase the accuracy of the CAM position data.

**Figure 2:**
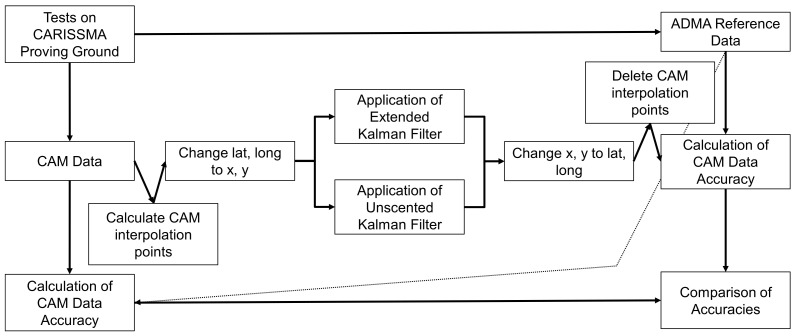
Extension of the methodological approach by temporarily adding additional CAM interpolation points to generate an equidistant time interval.

As the CAMs were sent at different time intervals of 0.1 to 1.0 s depending on the dynamics of the vehicle [5,29], it was also investigated whether the performance of the Kalman filters depends on this. To generate equidistant time intervals between the CAMs, imaginary linear interpolation points were generated between the CAMs at 0.1 and 0.01 s intervals. After applying the Kalman filters, the additional CAMs were deleted again, as illustrated in Figure 2. The accuracies were then also calculated and compared.

An alternative approach for addressing the unequal time intervals between the individual CAMs is the iterative adjustment of the Process Noise Covariance Matrix Q. As this is dependent on the time step Δt (see Section 3.1), the matrix Q was adapted to the time steps between the CAMs in a further investigation. Each time the time step between the CAMs changed, the Q matrix was recalculated with the current time step. This is understood in this work as an iterative adjustment or adaption of the Q matrix.

In addition to these investigations, two dynamic models were implemented and investigated in the Kalman filters. Table 1 summarizes all investigation parameters regarding the position accuracy of the CAM.

### 2.2. Experimental Set-Up and Design

**Figure 3:**
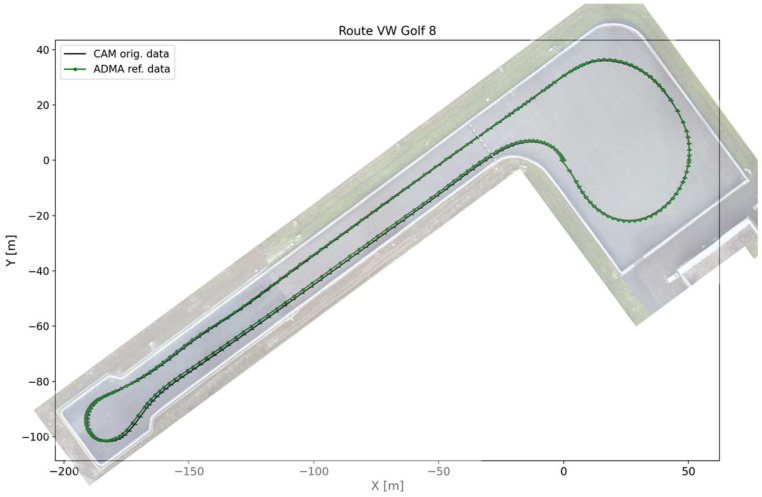
Top view of the CARISSMA outdoor test site with the trajectory of the test drives using the Golf 8 as an example.

The test data were generated at the CARISSMA outdoor test site at Technische Hochschule Ingolstadt, shown in Figure 3 from above.

The tests were carried out as part of the accuracy measurements published by the authors in [8]. Therefore, a detailed description of the received data and data structure, test vehicles, the measurement set-up, and the evaluation methods can be found in [8], and only a brief description is given below.

Two test vehicles were used to drive the trajectory on the test track shown in Figure 3. This was carried out three times with the Volkswagen Golf 8 and two times with the Volkswagen ID.3. As described in [8], both vehicles had an ADMA-G Pro+ [31] installed as the reference measurement system. The ADMA captures highly accurate inertial data as well as dGPS data. The CAMs of the test vehicles were received using a Commsignia Unit [32]. The CAM contains data on position, speed, longitudinal acceleration, heading, and yaw rate, which can be utilized for the Kalman filter. The expected accuracies depend on the dynamics of the vehicles and can be taken from [8]. Both data sets were transferred to a Robot Operating System (ROS) on the measurement computer and stored there. The two test vehicles’ technical data and software versions can also be found in [8].

In contrast to [8], these test drives generated a measurement from the combination of acceleration, deceleration, constant speed, and cornering to include all parts of a typical driving maneuver. This is intended to determine and demonstrate the general improvement in position accuracy using Kalman filters for everyday driving.

## 3. Modeling of Kalman Filters

### 3.1. Theoretical Background and Implementation of Extended Kalman Filter

Applying the simple Kalman filter is limited to linear problems, which does not apply to the description of general vehicle dynamics [33,34]. In this work, the Extended Kalman Filter (EKF) and the Unscented Kalman Filter were used to solve the problem, as these extend the classic Kalman filter to include non-linear state models.

**Figure 4:**
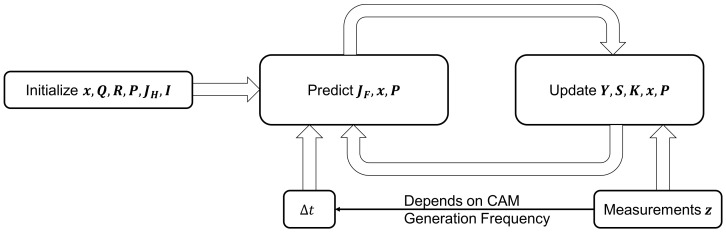
Schematic representation of the Kalman filter function according to [33].

Figure 4 shows schematically how the EKF works according to [33]. The EKF is initialized at the beginning. The state matrix x, process noise covariance matrix Q, sensor noise covariance matrix R, process covariance matrix P, the Jacobian matrix of the observation matrix $J_{H}$ and Identity Matrix I are defined according to the simulated system and filled with initial values for x and P. The state matrix x, which contains the available state parameters for describing the vehicle dynamics, must be defined first to dimension the matrices. The following parameters from CAM [5] can be used for this: Position data in latitude (lat) and longitude (long), the direction of travel (ψ), the longitudinal velocity (v), the yaw rate (ω) and the longitudinal acceleration (a). Since the data must be available in SI units for the correct system description, the position data were converted into global x and y coordinates of a Cartesian coordinate system with the origin in the first received CAM of the vehicle, according to [35,36].

This results in the following state matrix x:

(1) $$x={\left[x\;y\;\psi \;v\;\omega \;a\right]}^{T}$$

This means that the other matrices to be defined have six dimensions. During initialization, the first measurement vector $z_{0}$ of the measurement matrix Z was used for x, which corresponds to the data of the first CAM sent and received since the start of the measurement. In general, the measurements $z_{i}$ represent the individual CAMs sent by the vehicles and received and stored by the measuring equipment. The following applies to the initialization of x:

(2) $$x=z_{0}$$

The observation matrix H specifies which state vector x values are measured in the respective measurements $z_{i}$ [34]. Since the CAM always contains all data of the vector x, as these are mandatory parameters [5], and no non-linearities occur, the Jacobian matrix results in the observation matrix of the EKF:

(3) $$J_{H}=I(6)$$

To design the EKF specifically for the application of correcting position data with CAMs, the specific and adapted initialization of the noise matrices Q and R is necessary.

**Figure 5:**
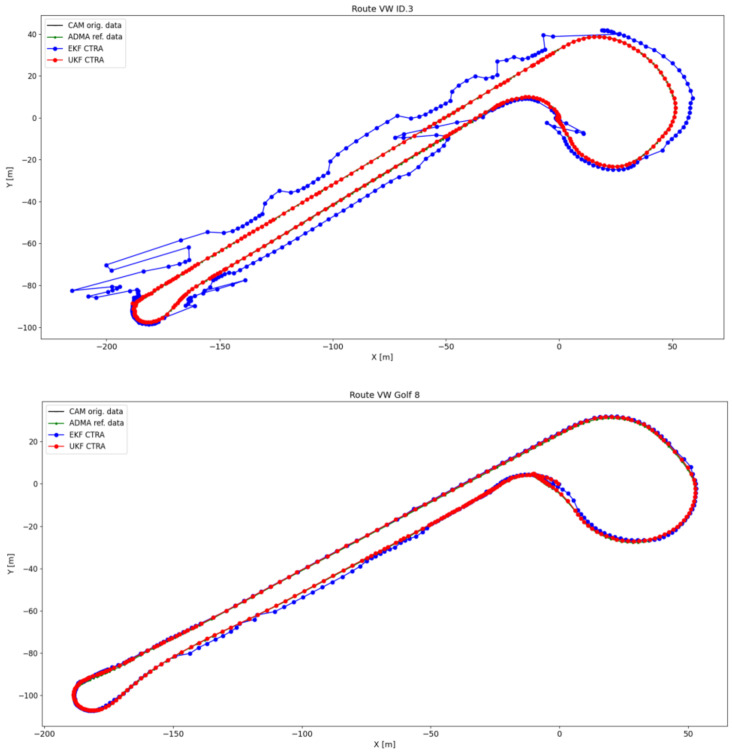
Presentation of the position information results after applying the Kalman filters. Top: Initialization of the R-matrix with heading in rad and yaw rate in rad/s. Bottom: Initialization of the R matrix with heading in ° and yaw rate in °/s.

The sensor noise covariance matrix R represents a diagonal matrix of the variances of the individual measured variables of the state vector x [34]. The state variables’ accuracy and standard deviation σ have already been determined by [8] and can be used directly to initialize R. In [8], standard deviations were determined for different dynamic states (accelerated travel, constant travel, cornering). Since this Kalman filter aims to estimate the CAM position data for any dynamic state, the mean value of the specified standard deviations was calculated and used. However, it must be added that the standard deviations for the heading and the yaw rate were not used in rad or rad/s but in ° and °/s. The reason for this is that the small entries in R made the Kalman gain K very large, which led to instabilities in the prediction, especially with the EKF, as illustrated in Figure 5 in blue on the left.

By squaring this value to calculate the variance, the following sensor noise covariance matrix was found:

(4) R=diag(5.917, 1.569, 35.16, 0.281, 19.36, 3.349)

In principle, any values could be used for the initialization of R, and R could also be part of the optimization in this work. However, since this is not the aim of this work and the initialization should be based on the actual measured values for the noise from [8], these values were chosen.

The Process Noise Covariance Matrix Q indicates how much the state variables change from iteration to iteration [33,34]. This matrix was also specially designed for use on CAMs and selected according to the trigger conditions [5]. The maximum possible change in the current state of motion results from the translational and rotational accelerations (a,ω) over the maximum possible time step Δt.

From the trigger conditions of the CAM, it can be deduced that a maximum frequency of 10 Hz occurs at an acceleration $a=5.0\;m/s^{2}$ and a yaw rate of ω=0.70 rad/s. The maximum possible time step is Δt=1.0 s.

This results in the following process variables using simple kinematic relationships:

(5) $$\left[\begin{array}{c} dx\\ dy\\ \begin{array}{c} d\psi \\ dv\\ \begin{array}{c} d\omega \\ da \end{array} \end{array} \end{array}\right]=\left[\begin{array}{c} 0.5\;a\;\Delta t^{2}\\ 0.5\;a\;\Delta t^{2}\\ \begin{array}{c} \omega \;\Delta t\\ a\;\Delta t\\ \begin{array}{c} \omega \\ a \end{array} \end{array} \end{array}\right]$$

Using the above values in the formulas and then squaring the results in the following Process Noise Covariance Matrix gives the following:

(6) Q=diag(6.250, 6.250, 0.487, 25.00, 0.487, 25.00)

When initializing a Kalman filter, the process covariance matrix P must be defined, which describes the uncertainty of the initial state estimate [33]. High initial values for P imply that the initial state estimate contains a high level of uncertainty. Therefore, the measured values (CAMs) have a greater weight in the state estimate at the start of the iterations between prediction and update [33]. To ensure that the CAMs are weighted more heavily at the beginning, P was selected as a diagonal matrix with 3σ of the individual measured variables in accordance with [30]:

(7) P=diag(7.298, 3.758, 17.79, 1.590, 13.20, 5.490)

Finally, the state transition matrix $J_{F}$ must be determined from the state transition function vector f in relation to the state vector ***x***. The Constant Turn Rate and Acceleration (CTRA) model is used for the general description of vehicle dynamics, as it describes the vehicle’s driving kinematics considering all available state variables [28]. Since the angle ψ describes the heading of the vehicle in a mathematically negative direction starting from the global y-axis (north vector) [5], ψ is transformed into the mathematical positive direction starting from the global x-axis for each CAM:

(8) ψ=(360°−ψ)+90°

Subsequently, the following applies to f for the CTRA Model if ω≠0:

(9) $$f=\left[\begin{array}{c} x_{t+1}\\ y_{t+1}\\ \begin{array}{c} \psi_{t+1}\\ v_{t+1}\\ \begin{array}{c} \omega_{t+1}\\ a_{t+1} \end{array} \end{array} \end{array}\right]=\left[\begin{array}{c} x_{t}+\frac{v_{t}}{\omega_{t}}\left(\sin\left(\psi_{t}+\omega_{t}\Delta t\right)-\sin\left(\psi_{t}\right)\right)+\frac{a_{t}}{\omega_{t}^{2}}(\cos\left(\psi_{t}\right)-\cos\left(\psi_{t}+\omega_{t}\Delta t\right))\\ y_{t}+\frac{v_{t}}{\omega_{t}}\left(-\cos\left(\psi_{t}+\omega_{t}\Delta t\right)+\cos\left(\psi_{t}\right)\right)+\frac{a_{t}}{\omega_{t}^{2}}(-\sin\left(\psi_{t}+\omega_{t}\Delta t\right)+\sin(\psi_{t}))\\ \begin{array}{c} \psi_{t}+\omega_{t}\Delta t\;\\ v_{t}+a_{t}\Delta t\\ \begin{array}{c} \omega_{t}\\ a_{t} \end{array} \end{array} \end{array}\right]$$

If ω=0, the system simplifies itself:

(10) $$f=\left[\begin{array}{c} x_{t+1}\\ y_{t+1}\\ \begin{array}{c} \psi_{t+1}\\ v_{t+1}\\ \begin{array}{c} \omega_{t+1}\\ a_{t+1} \end{array} \end{array} \end{array}\right]=\left[\begin{array}{c} x_{t}+v_{t}\cos\left(\psi_{t}\right)\Delta t+\frac{1}{2}a_{t}\cos\left(\psi_{t}\right)\Delta t\\ x_{t}+v_{t}\sin\left(\psi_{t}\right)\Delta t+\frac{1}{2}a_{t}\sin\left(\psi_{t}\right)\Delta t\\ \begin{array}{c} \psi_{t}\\ v_{t}+a_{t}\Delta t\\ \begin{array}{c} 0\\ a_{t} \end{array} \end{array} \end{array}\right]$$

In practice, the condition ω=0 leads to instabilities and errors in the prediction. This is because ω=0 does not occur even when driving straight ahead, as it only becomes tiny and, therefore, the simplified model is never addressed. Since ω is in the denominator in the CTRA model, the speed and acceleration are weighted very heavily, leading to prediction errors. In this work, a study was conducted to find a good limit value for ω for switching between the CTRA and simplified model (see Section 3.1). The threshold for ω to switch between the full and simplified model was systematically performed from 0.00 to 0.50 rad/s in 0.05 rad/s steps, and the resulting medians, means, and standard deviations for dx and dy were analyzed concerning the reference without Kalman filter. For this purpose, a metric was introduced which, starting from the position accuracy without applied Kalman filter ($dx_{CAM},\;dy_{CAM}$), gives the respective result from the Kalman filters (extended and unscented) for $dx_{KF}$ and $dy_{KF}$ a value between 0 (no improvement or deterioration in accuracy) and 100 (no more inaccuracy). For the calculation rule, if $\left|dx_{KF}\;|<|dx_{CAM}\;\right|$ and $\left|dy_{KF}\;|<|dy_{CAM}\;\right|$ applies,

(11) $$Value_{dx}=\frac{\left|dx_{CAM}|-|dx_{KF}\right|}{\left|dx_{CAM}\right|},Value_{dy}=\frac{\left|dy_{CAM}|-|dy_{KF}\right|}{\left|dy_{CAM}\right|}$$

The Jacobi matrix is then calculated from the partial derivatives of the state transition function vector f with respect to the state vector f at time t.

(12) $$J_{F}\left(t\right)=\frac{\partial f}{\partial x}(t)$$

The calculation rules within the predict and update method follow the classic calculation rule of the Kalman filter. The calculation rules and the formal procedure can be found in [33,34] and are not explicitly shown here.

### 3.2. Extended Kalman Filter Including Sideslip Angle

An Extended Kalman Filter was presented in [30], considering the sideslip angle beta (β) in its kinematic model, which improved position accuracy. As this was also the aim of this work about the CAM, the motion model from [30], referred to as the SSA model in the following, was also implemented and compared with the results of the CTRA model. The state vector x thus changes to

(13) $$x={\left[x\;y\;\psi \;\beta \;v\;\omega \;a\right]}^{T}$$

According to [30], the state transition function vector f for v>1.5 m/s results in

(14) $$f=\left[\begin{array}{c} x_{t+1}\\ y_{t+1}\\ \begin{array}{c} \begin{array}{c} \psi_{t+1}\\ \beta_{t+1} \end{array}\\ v_{t+1}\\ \begin{array}{c} \omega_{t+1}\\ a_{t+1} \end{array} \end{array} \end{array}\right]=\left[\begin{array}{c} x_{t}+v_{t}\cos\left(\psi_{t}+\beta_{t}\right)\Delta t+\frac{a_{t}}{2}\cos\left(\psi_{t}\right)\Delta t^{2}\\ y_{t}+v_{t}\sin\left(\psi_{t}+\beta_{t}\right)\Delta t+\frac{a_{t}}{2}\sin\left(\psi_{t}\right)\Delta t^{2}\\ \begin{array}{c} \psi_{t}+\omega_{t}\Delta t\\ \arctan\left(\frac{l\omega_{t}}{v}\right)\\ \begin{array}{c} v_{t}+a_{t}\Delta t\\ \omega_{t}\\ a_{t} \end{array} \end{array} \end{array}\right]$$

and simplifies at v<1.5 m/s to

(15) $$f=\left[\begin{array}{c} x_{t+1}\\ y_{t+1}\\ \begin{array}{c} \begin{array}{c} \psi_{t+1}\\ \beta_{t+1} \end{array}\\ v_{t+1}\\ \begin{array}{c} \omega_{t+1}\\ a_{t+1} \end{array} \end{array} \end{array}\right]=\left[\begin{array}{c} x_{t}+v_{t}\cos\left(\psi_{t}+\beta_{t}\right)\Delta t+\frac{a_{t}}{2}\cos\left(\psi_{t}\right)\Delta t^{2}\\ y_{t}+v_{t}\sin\left(\psi_{t}+\beta_{t}\right)\Delta t+\frac{a_{t}}{2}\sin\left(\psi_{t}\right)\Delta t^{2}\\ \begin{array}{c} \psi_{t}+\omega_{t}\Delta t\\ 0\\ \begin{array}{c} v_{t}+a_{t}\Delta t\\ \omega_{t}\\ a_{t} \end{array} \end{array} \end{array}\right]$$

As the dimension increases by 1, the matrices Q,P,R,H and I must also be adjusted accordingly. The standard deviation of the sideslip angle was set to 0.009 rad/s according to [37] for small sideslip angles. A maximum possible sideslip angle of 0.35 rad was assumed for Q accordingly.

(16) $$Q=diag(6.250,\;6.250,\;0.487,\;0.122,\;25.00,\;0.487,\;25.00)\;P=diag(7.298,\;3.758,\;17.79,\;0.026,\;1.590,\;13.20,\;5.490)\;R=diag(5.917,\;1.569,\;35.16,\;7.615\;e-05,\;0.281,\;19.36,\;3.349)\;J_{H}=diag(1,\;1,\;1,\;0,\;1,\;1,\;1)\;I=I(7)$$

### 3.3. Unscented Kalman Filter

Compared to the Extended Kalman Filter (EKF), the Unscented Kalman Filter (UKF) adopts a fully non-linear approach to calculate the state variables from the input variables [37]. The EKF approximates non-linear systems by linearizing them using the Jacobian matrices of the system and measurement models. This linearization process can introduce errors if the system exhibits significant non-linear behavior, as the EKF assumes that the first-order Taylor expansion adequately captures the system dynamics.

In contrast, the UKF eliminates the need for linearization by employing a deterministic sampling technique. It uses so-called “sigma points,” strategically chosen around the mean of the state distribution as a function of the standard deviations. These sigma points are then propagated through the non-linear system dynamics and measurement models, capturing the true non-linear transformations. The UKF subsequently uses these transformed sigma points to compute the mean and covariance of the predicted states. This approach allows the UKF to maintain a higher accuracy in capturing the effects of non-linearities compared to the EKF [37].

Additionally, the UKF often shows superior performance when dealing with highly non-linear systems, particularly in scenarios where the Jacobian matrices required by the EKF are difficult to compute or are numerically unstable. However, this increased accuracy comes at the cost of slightly higher computational complexity due to the propagation of multiple sigma points. The mathematical formulations for both methods, including the generation and propagation of sigma points in the UKF, can be found in [37].

When implementing the UKF, the same system matrices must be defined analogously to the initialization of the EKF. The kinematic models must also be implemented in the same way.

Both Kalman filters (EKF and UKF) were implemented in Python for this work. The EKF was self-programmed using the numpy and pandas library [38,39]. The UKF was implemented using the filterpy library [40]. This made it possible to apply the UKF directly to the measurement data.

## 4. Results

### 4.1. Application Analysis of Kalman Filters and Kinematic Model

**Figure 6:**
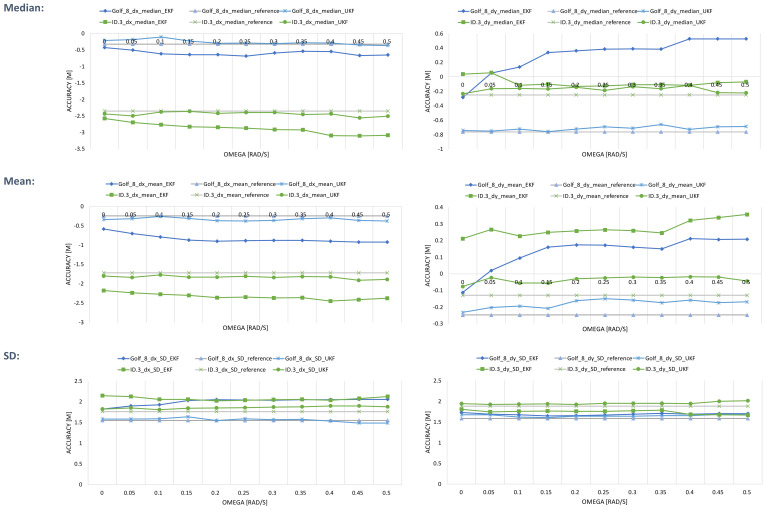
Result of the ω-analysis: progression of median, mean, and standard deviation via omega. In green: Filtered position data (EKF and UKF) of the ID.3 with CTRA model. In blue: Filtered position data (EKF and UKF) of the Golf 8 with CTRA model. (SD = standard deviation).

As mentioned in Section 3.1, an analysis was conducted at the beginning of the work to define an ω limit value to determine the change from the full to the simplified CTRA model. Figure 6 shows the analysis results for the median, mean, and standard deviation of the longitudinal and lateral position accuracy dx and dy over ω. Please note that dx and dy are always determined from the point of view of the vehicle’s local coordinate system, depending on the direction of travel.

The left column shows the diagrams for dx and the right column for dy. The result for the VW Golf 8 is shown in blue, and VW ID.3 in green. Each diagram has three curves of accuracy over ω for each vehicle. Two of them show the accuracy of dx and dy when using the EKF or UKF with the CTRA model at the respective ω limit value. The corresponding accuracies without the Kalman filter applied, which serve as a reference, are shown in gray. The closer the points are to 0, the higher the accuracy and the better the result. However, it is not immediately apparent from the curves for the accuracy of the two vehicles in the median, mean value, and standard deviation which ω limit value is best for use in the Kalman filter. In principle, the standard deviation after applying the Kalman filter is always higher than the reference and does not change significantly over ω. Regarding the longitudinal position accuracy (dx), it can be seen that the application of the Kalman filter only improves the accuracy for the Golf 8 for small ω in combination with the UKF but worsens it for the ID.3. In contrast, the lateral position accuracy (dy) is improved by the Kalman filter for both vehicles for certain ω limit values.

A second analysis was conducted using Equation (11) to identify the optimum ω limit for further analyses. Table 2 shows the result of the calculation rule and the sum of the scores achieved for each ω limit value. A color gradient from red (minimum value) to green (maximum value) is then shown in each summary row to enable a visual analysis of the performance of the Kalman filters per ω limit value.

In addition to the total sum, the scores for various parameters were evaluated separately to specify further the Kalman filters’ performance at various ω limit values. For example, the Golf 8 and ID.3 scores were evaluated separately. This shows that the best ω limit value for the ID.3 (ω=0.4) is one of the worst for the VW Golf 8. Different results can also be seen when looking at dx and dy separately. Even if different “best” ω limit values are obtained for the accuracies of dx and dy as well as for the Golf 8 and ID.3, it is also clear that there is an ω limit value that delivers good performance for all of them. For this reason, an ω limit value of 0.05 was defined for further analyses.

**Figure 7:**
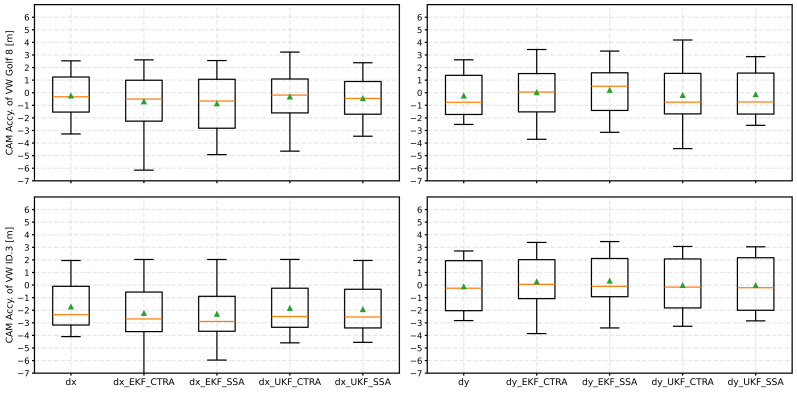
Result of the position accuracies as a boxplot diagram after applying the various Kalman filters (EKF and UKF) with the respective kinematic models. The median is an orange line, and the mean value is a green triangle.

Figure 7 shows a boxplot diagram summarizing the accuracies of the CAM position data after applying EKF and UKF with both the CTRA and SSA models. The orange line represents the median, and the green triangle represents the mean value.

The two diagrams on the left show the accuracies of the longitudinal position (dx) for the two test vehicles. For the EKF, it can generally be seen that the average accuracy (median and mean value) deteriorates compared to the accuracy without a filter. At the same time, a higher scattering can be observed in the box plots. When looking at the UKF, on the other hand, the median accuracy improves with the CTRA model for Golf 8, while the mean value remains roughly the same. With the ID.3, only a minimal deterioration can be recognized with the CTRA model. The dispersion of the values is also smaller than with the EKF model. The SSA model also delivers worse results for dx with the UKF than the CTRA model. However, these are better with the UKF than with the EKF.

Regarding the lateral position accuracy (dy), the EKF shows the best results with the CTRA model. The median here is close to zero for both test vehicles. In contrast to dx, the EKF performs better this time than the UKF. As with dx, the CTRA model delivers better results than the SSA model.

### 4.2. Iterative Q Matrix Adjustment

Based on the results so far, it can be observed that the accuracy could not be significantly improved by using the Kalman filters. This could be due to the difficulties already mentioned.

**Figure 8:**
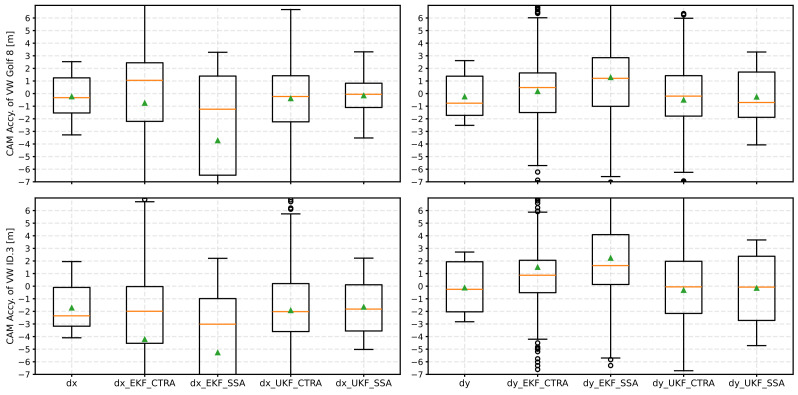
Result of the position accuracies as a boxplot diagram after applying the various Kalman filters (EKF and UKF) with the respective kinematic models and the Q matrix adjustment. The median is an orange line, and the mean value is a green triangle.

To counter this problem, an iterative adjustment of the Q matrix was implemented and examined when applying Kalman filters to CAM data. The result can be found in Figure 8.

The EKF has massive problems with the iterative adjustment of the Q-matrix. In addition to the large scatter and general deterioration of the determined accuracies, the mean values and medians are far from each other. This shows that the filter tends to be unstable due to the measure and leads no longer to reasonable results. In contrast, the UKF performs more stable with the iterative Q-matrix adjustment. The SSA model, in particular, shows an improved accuracy of both position data for both vehicles in the median. In addition, the scatter is of a comparable order of magnitude to the reference without the Kalman filter applied.

### 4.3. Equidistantiation with Linear Interpolation Points

As a further approach, the introduction of imaginary linear interpolation points was investigated. Here, the time intervals were adjusted to Δt=0.1 s in accordance with the CAM’s maximum generation frequency of 10 Hz [5] and to Δt=0.01 s (100 Hz) in a further investigation.

**Figure 9:**
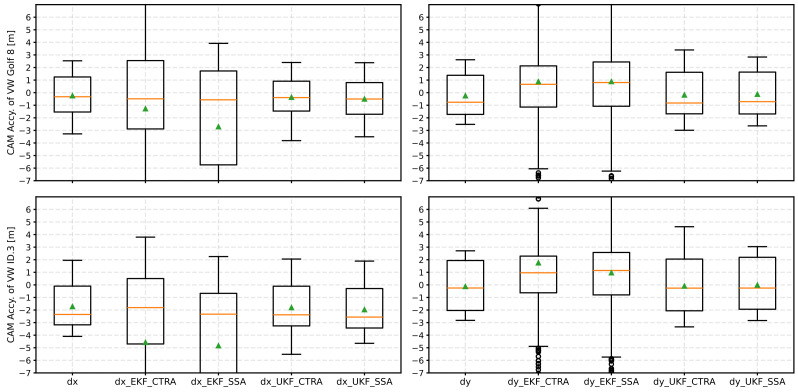
Result of the position accuracies as a boxplot diagram after applying the various Kalman filters (EKF and UKF) with the respective kinematic models and after equidistancing to Δt=0.1 s. The median is shown as an orange line and the mean value as a green triangle.

Figure 9 illustrates the result with equidistant time steps of 0.1 s. Again, the EKF tends to be unstable and shows a large scatter and deterioration of the mean accuracies. In contrast, the UKF is more stable. However, a significant improvement in accuracy could not be determined for any kinematic model with UKF. Instead, it changed only slightly compared to the reference for both dx and dy.

**Figure 10:**
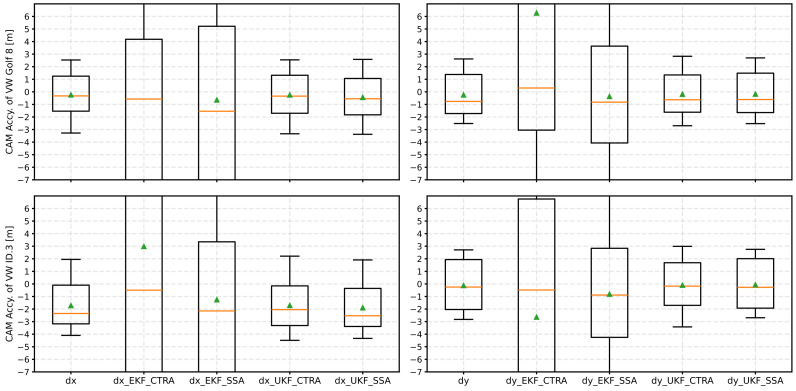
Result of the position accuracies as a boxplot diagram after applying the various Kalman filters (EKF and UKF) with the respective kinematic models and after equidistancing to Δt=0.01 s. The median is shown as an orange line and the mean value as a green triangle.

Finally, Figure 10 presents the result when adding linear interpolation points for a time interval of Δt=0.01 s.

The instability of the EKF continues to increase for both kinematic models. In contrast, the UKF remains stable again. However, as with the equidistant time interval of 0.1 s, the results show only a slight change compared to the reference. For the UKF with the CTRA model, a slight median improvement can be seen for both vehicle position data sets.

### 4.4. Results Summary of the Best Approaches

The following compares the best approaches from the three sections above. Section 4.1 shows that the UKF with the CTRA model performed best for dx. For the improvement of dy, on the other hand, the EKF with the CTRA model was better. In Section 4.2, the UKF with the SSA model delivered the best mean accuracy for dx and dy for both vehicles. In Section 4.3, this was the case for the UKF with CTRA model at a time interval of Δt=0.01 s.

Table 3 lists the medians, mean values, and standard deviations of the respective approaches compared to the reference. It can be seen here again that the Kalman filter’s plane application to the CAM data leads to different results depending on the vehicle and position data set. This could be because of the difficulties mentioned earlier due to the low temporal resolution and non-equidistant time intervals between the CAMs.

As the median is less sensitive to outliers, it was used for further analysis. In Table 4, the absolute difference between the median reference values and the median values using the respective approach (ΔMedian) was calculated. In addition, the percentage change from the reference value was calculated and presented (Δ%Median).

By using the Q matrix adjustment or introducing additional interpolation points, it was possible to create a UKF that improves median position accuracy for both vehicles. The combination of Q matrix adjustment with UKF and SSA model shows the best results from all investigated approaches. The longitudinal accuracy could be improved by a median value of between 0.26 and 0.54 m, corresponding to an increase in accuracy of between 23.0% and slightly over 80%. The lateral position accuracy could be improved with this approach between 0.05 m and 0.18 m, which corresponds to an improvement of 6.58 to 72.0%.

**Figure 11:**
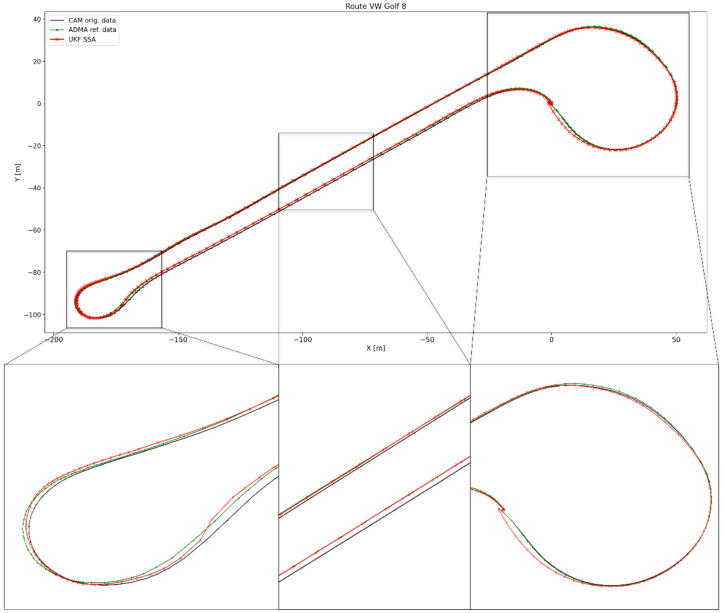
Plot of the position points of the driven trajectory according to the original CAM data (black), the ADMA reference data (green), and the position data after applying the UKF with SSA model and Q-matrix adjustment (red).

Figure 11 illustrates in an XY diagram the position points according to the original CAM data (black), ADMA reference data (green), and the CAM data after these have been filtered by the UKF with SSA model and Q matrix adjustment (red). The two curves and the straight section in the middle of the route are also shown in a zoomed view. Overall, when looking at the position points of the filtered data over the entire route, a plausible trajectory without sudden jumps between the position points can be observed. On closer inspection, it is noticeable that the filter improves the lateral positioning accuracy when driving straight ahead and is almost congruent with the reference data.

When cornering in the left zoom image, this congruence is resolved. The filtered position points are mostly between the original CAM data and the ADMA reference data, showing that the filter improves the lateral position accuracy when cornering. After exiting the curve, the filtered data are again aligned with the ADMA reference data.

At the start of the right curve run, the filtered data are slightly further away from the reference data than the original CAM position data for the first time. However, as the curve progresses, the position points approach the reference data again until all three are congruent. A slightly larger deviation from the reference data can be seen at the end of the curve until the filtered data and ADMA reference data meet again at the last data point.

## 5. Discussion

This study explored the application of Kalman filters to Cooperative Awareness Message (CAM) position data to improve positional accuracy in post-processing. The key challenges addressed were the CAM data points’ low temporal resolution and non-equidistant time intervals. Extended Kalman Filters (EKF) and Unscented Kalman Filters (UKFs) were designed using different kinematic models, including Constant Turn Rate and Acceleration (CTRA) and sideslip angle (SSA), to assess their effectiveness in addressing these challenges.

The results demonstrate that EKF and UKF can enhance positional accuracy under specific conditions. Specific filter configurations, such as the EKF with the CTRA model, were found to have superior corrections for lateral position accuracy in specific scenarios. However, this improvement often came at the cost of worsened longitudinal position accuracy. Only the SSA model paired with UKF provided the most consistent improvements, especially after iterative adjustments to the Process Noise Covariance Matrix Q, which depends on the time intervals between CAMs. This at least partially confirms the results from [28] that improving the accuracy of CAM position data with a Kalman filter based only on CAM data is possible. However, the filters’ performance was inconsistent across vehicles. While improvements were observed for one vehicle’s position accuracy, the same filter configuration often degraded accuracy for another. Similar inconsistencies were noted in the individual position components dx and dy, emphasizing the need to refine the filter models further.

One potential way to further enhance the performance of the Kalman filters under investigation would be to incorporate lateral acceleration. Since the data in the CAM were optional and this information was not included in our measurements, they were unavailable for this study. However, information about lateral acceleration could significantly improve the performance of the developed Kalman filter, particularly during cornering and directional changes, as it would allow for a more accurate estimation of the driving direction. Additionally, lateral acceleration could be used to validate the consistency of the current speed and yaw rate, as these parameters are directly mathematically correlated. This would, in turn, increase the robustness of the developed filters during cornering maneuvers.

The low temporal resolution and non-equidistant CAM intervals likely exacerbated the inconsistencies, as the Q matrix relies on accurate time-step estimation. Linear interpolation to equidistant CAM points did not yield significant improvements and may have introduced errors from the linearized points that misled the filters.

Initializing the sensor noise covariance matrix R also posed challenges. Based on prior measurement results, this matrix included measurement noise for heading and yaw rate in degrees rather than radians, which could have affected filter performance. This indicates a need for further optimization of the Kalman filter design.

The filters showed promising results for applications like accident analysis and ETC, particularly in achieving centimeter-level accuracy for lateral positioning. However, longitudinal positioning accuracy was problematic, with the initial inaccuracies of over 2 m for certain vehicles persisting despite filter application. These findings highlight the potential of Kalman filters for improving CAM data accuracy but also reveal limitations that require further investigation.

## 6. Conclusions

This work contributes to the literature by systematically addressing the challenges of applying Kalman filters to Cooperative Awareness Message (CAM) data for post-processing accuracy enhancements. Unlike prior studies, this research focused exclusively on filters that rely solely on the data contained within CAMs, without external sources, and applied them to real driving test data from commercially available vehicles. This is the first study to investigate the feasibility and performance of such an approach under realistic conditions, offering novel insights into filter design and parameterization.

Both Extended Kalman Filters (EKFs) and Unscented Kalman Filters (UKFs) were created using kinematic models such as Constant Turn Rate and Acceleration (CTRA) and sideslip angle (SSA). Among these, the UKF combined with iterative adjustments to the Process Noise Covariance Matrix Q provided the most consistent results, achieving significant improvements in positional accuracy. Quantitatively, the adapted UKF enhanced the longitudinal position accuracy of the two vehicles investigated by up to 80% (0.54 m) and lateral position accuracy by up to 72% (0.18 m). These results underscore the efficacy of the proposed filter design in addressing the challenges posed by low temporal resolution and non-equidistant intervals in CAM data.

However, the findings also highlight limitations. The longitudinal inaccuracies of certain vehicles, especially those with initial deviations exceeding 2 m, could not be fully compensated by any filter configuration. Additionally, linear interpolation to equidistant CAM points did not significantly improve accuracy; instead, errors introduced by interpolation likely counteracted potential benefits. Furthermore, initializing the sensor noise covariance matrix R in degrees rather than radians highlighted areas for further refinement in the filter design.

The filters designed here are promising for lateral positioning tasks, achieving centimeter-level accuracy. Integrating Kalman-filter-enhanced data with traditional reconstruction methods is recommended for accident reconstruction and analysis. This dual approach ensures robust validation of CAM-based analyses and expands their practical applicability.

## Figures and Tables

**Table 1:** Summary of the investigations to create a Kalman filter for the Cooperative Awareness Message.

Research Subject	Description
Kalman Filter	Application of Extended Kalman Filter and Unscented Kalman Filter
Kinematic model	Application of CTRA and dynamic model with sideslip angle according to [30] ω-Analysis of CTRA model
Iterative Q matrix adjustment	Influence analysis of iterative Q matric adjustment
Linear interpolation points	Influence analysis of linear interpolation points (Δt=0.1 and 0.01 s)

**Table 2:** Result of the ω analysis: quantification of the results for median, mean, and standard deviation according to Equation (11). The worst (red) result and the best (green) result are indicated by a gradation from red to yellow to green.

Approach ↓/omega →	0.00	0.05	0.10	0.15	0.20	0.25	0.30	0.35	0.40	0.45	0.50
median EKF CTRA	0.00	0.00	0.00	0.00	0.00	0.00	0.00	0.00	0.00	0.00	0.00
mw EKF CTRA	0.00	0.00	0.00	0.00	0.00	0.00	0.00	0.00	0.00	0.00	0.00
SD EKF CTRA	0.00	0.00	0.00	0.00	0.00	0.00	0.00	0.00	0.00	0.00	0.00
median UKF CTRA	33.5	41.7	65.7	27.5	7.80	10.9	5.04	11.8	9.27	0.00	0.00
mw UKF CTRA	0.00	0.00	0.00	0.00	0.00	0.00	0.00	0.00	0.00	0.00	0.00
SD UKF CTRA	0.00	0.00	0.00	0.00	0.04	0.00	0.00	0.00	0.64	3.65	3.69
median EKF CTRA	62.6	92.9	82.1	55.5	52.8	49.7	48.9	49.4	31.1	31.1	31.1
mw EKF CTRA	54.8	92.3	61.2	35.2	29.8	30.5	35.4	39.4	14.5	16.7	16.1
SD EKF CTRA	0.00	0.00	0.00	0.00	0.00	0.00	0.00	0.00	0.00	0.00	0.00
median UKF CTRA	2.39	1.51	4.80	0.54	5.20	8.99	6.29	13.4	4.66	9.22	9.68
mw UKF CTRA	6.42	18.1	21.4	15.7	34.8	39.5	35.8	29.9	36.0	29.8	31.8
SD UKF CTRA	0.00	0.00	0.00	0.00	0.00	0.00	0.00	0.00	0.00	0.00	0.00
median EKF CTRA	0.00	0.00	0.00	0.00	0.00	0.00	0.00	0.00	0.00	0.00	0.00
mw EKF CTRA	0.00	0.00	0.00	0.00	0.00	0.00	0.00	0.00	0.00	0.00	0.00
SD EKF CTRA	0.00	0.00	0.00	0.00	0.00	0.00	0.00	0.00	0.00	0.00	0.00
median UKF CTRA	0.00	0.00	0.00	0.00	0.00	0.00	0.00	0.00	0.00	0.00	0.00
mw UKF CTRA	0.00	0.00	0.00	0.00	0.00	0.00	0.00	0.00	0.00	0.00	0.00
SD UKF CTRA	0.00	0.00	0.00	0.00	0.00	0.00	0.00	0.00	0.00	0.00	0.00
median EKF CTRA	85.5	77.3	54.0	59.0	46.8	50.5	55.7	57.0	53.5	68.2	71.9
mw EKF CTRA	0.00	0.00	0.00	0.00	0.00	0.00	0.00	0.00	0.00	0.00	0.00
SD EKF CTRA	4.29	7.50	6.60	6.53	6.66	6.64	6.15	5.22	93.9	11.1	11.4
median UKF CTRA	6.43	35.0	36.3	32.3	42.3	25.2	45.3	34.0	53.7	11.3	11.2
mw UKF CTRA	40.6	82.6	56.6	57.6	76.8	81.5	84.4	82.9	86.4	85.2	66.0
SD UKF CTRA	0.00	0.00	0.00	0.00	0.00	0.00	0.00	0.00	0.00	0.00	0.00
SUM All	297	449	389	290	303	303	323	323	384	266	253
SUM Median	190	248	243	175	155	145	161	166	152	120	124
SUM Mean	102	193	139	109	141	152	156	152	137	132	114
SUM EKF	207	270	204	156	136	137	146	151	193	127	131
SUM UKF	89.4	179	185	134	167	166	177	172	191	139	122
SUM dx	33.5	41.7	65.7	27.5	7.85	10.9	5.04	11.8	9.91	3.65	3.69
SUM dy	263	407	323	262	295	293	318	311	374	263	249
SUM Golf 8	160	246	235	135	130	140	131	144	96.2	90.5	92.4
SUM ID.3	137	202	153	155	173	164	191	179	287	176	161

**Table 3:** Results comparison of the best Kalman filter configurations from the different studies.

Golf 8:	Median	Mean	SD	ID.3:	Median	Mean	SD
Reference:							
dx	−0.32 m	−0.24 m	1.54 m		−2.35 m	−1.72 m	1.76 m
dy	−0.76 m	−0.25 m	1.58 m		−0.25 m	−0.13 m	1.88 m
Section 3.1: Plane KF Application							
dx_UKF_CTRA	−0.19 m	−0.32 m	1.58 m		−2.50 m	−1.84 m	1.85 m
dy_UKF_CTRA	−0.75 m	−0.20 m	1.67 m		−0.16 m	−0.02 m	1.92 m
dx_EKF_CTRA	−0.50 m	−0.71 m	1.90 m		−2.69 m	−2.24 m	2.12 m
dy_EKF_CTRA	0.05 m	0.02 m	1.69 m		0.06 m	0.27 m	1.74 m
Section 3.2: Q-Matrix Adjustment							
dx_UKF_SSA	−0.06 m	−0.16 m	1.46 m		−1.81 m	−1.65 m	2.01 m
dy_UKF_SSA	−0.71 m	−0.27 m	1.98 m		−0.07 m	−0.16 m	2.51 m
Section 3.3: Equidistantiation							
dx_UKF_CTRA_0.01	−0.34 m	−0.25 m	1.68 m		−2.05 m	−1.71 m	1.80 m
dy_UKF_CTRA_0.01	−0.63 m	−0.19 m	1.51 m		−0.17 m	−0.10 m	1.77 m

**Table 4:** Result of the absolute and relative difference between the median reference values without Kalman filters and the respective analyzed approaches. Improvements in position accuracy are shown in green, deteriorations in red.

Golf 8:	Median	ΔMedian	Δ%Median	ID.3:	Median	ΔMedian	Δ%Median
Section 3.1: Plane KF Application							
dx_UKF_CTRA	−0.19 m	▲0.13 m	▲40.6%		−2.50 m	▼0.15 m	▼6.38%
dy_UKF_CTRA	−0.75 m	▲0.01 m	▲1.32%		−0.16 m	▲0.09 m	▲36.0%
dx_EKF_CTRA	−0.50 m	▼0.18 m	▼56.3%		−2.69 m	▼0.34 m	▼14.5%
dy_EKF_CTRA	+0.05 m	▲0.71 m	▲93.4%		0.06 m	▲0.19 m	▲76.0%
Section 3.2: Q-Matrix Adjustment							
dx_UKF_SSA	−0.06 m	▲0.26 m	▲81.3%		−1.81 m	▲0.54 m	▲23.0%
dy_UKF_SSA	−0.71 m	▲0.05 m	▲6.58%		−0.07 m	▲0.18 m	▲72.0%
Section 3.3: Equidistantiation							
dx_UKF_CTRA_0.01	−0.34 m	▼0.02 m	▼6.25%		−2.05 m	▲0.30 m	▲12.8%
dy_UKF_CTRA_0.01	−0.63 m	▲0.13 m	▲17.1%		−0.17 m	▲0.08 m	▲32.0%

## Data Availability

The data presented in this study are available on request from the corresponding author.
